# Correlation of Sleep Duration and Sleep Quality With Coronary Atherosclerotic Plaque Vulnerability: An Optical Coherence Tomography Study

**DOI:** 10.31083/RCM41098

**Published:** 2025-11-12

**Authors:** Qingbo Shi, Yang Gao, Zhiwen Zhang, Zhuocheng Shi, Haosen Yu, Tong Zhang, Mingxing Lv, Donghui Chen, Zhenzhou Zhao, Yushuo Gu, Quan Guo, Cao Ma, Muwei Li

**Affiliations:** ^1^Department of Cardiology, Central China Fuwai Hospital of Zhengzhou University, Fuwai Central China Cardiovascular Hospital, 450000 Zhengzhou, Henan, China; ^2^Department of Cardiology, Zhengzhou University People's Hospital, Henan Provincial People's Hospital, 450000 Zhengzhou, Henan, China; ^3^Central China Subcenter of National Center for Cardiovascular Diseases, Henan Cardiovascular Disease Center, 450000 Zhengzhou, Henan, China; ^4^Henan Provincial Clinical Research Center for Cardiovascular Disease, 450000 Zhengzhou, Henan, China

**Keywords:** sleep duration, sleep quality, thin-cap fibroatheroma, plaque vulnerability, optical coherence tomography

## Abstract

**Background:**

Previous studies have shown a strong link between sleep and cardiovascular disease. However, the association of sleep duration and quality with coronary atherosclerotic plaque vulnerability remains unclear. This study aimed to investigate the correlation between sleep duration, sleep quality, and coronary plaque vulnerability using optical coherence tomography (OCT).

**Methods:**

A total of 260 patients with stable angina who completed an OCT examination were included. Patients were divided into a thin-cap fibroatheroma (TCFA) group and a non-TCFA group according to the presence of TCFA on OCT. The sleep duration of the patients was recorded by questionnaire, and the sleep quality was evaluated using the Pittsburgh Sleep Quality Index (PSQI).

**Results:**

The TCFA group had significantly shorter sleep duration and higher PSQI values (*p* < 0.05). A multivariable logistic regression analysis revealed that sleep duration and PSQI were independent predictors of TCFA (*p* < 0.05). A receiver operating characteristic (ROC) study demonstrated that the area under the curve values for sleep duration and PSQI were 0.698 and 0.721, respectively, in predicting the presence of TCFA. Patients with a sleep duration ≤5.5 hours or a PSQI value >9 had a thinner fibrous cap thickness, a larger maximal lipid pool arc, and a higher incidence of TCFA and macrophage deposition (*p* < 0.05). Sleep duration was positively correlated with the thinnest fibrous cap thickness (r = 0.451; *p* < 0.001), and negatively correlated with the radian of the maximum lipid pool (r = –0.470; *p* < 0.001). The PSQI was negatively correlated with the thinnest fibrous cap thickness (r = –0.477; *p* < 0.001), and positively correlated with the radian of maximum lipid pool (r = 0.340; *p* < 0.001).

**Conclusion:**

Both sleep duration and sleep quality were significantly associated with coronary plaque vulnerability. Patients with either insufficient sleep duration or poor sleep quality exhibited significantly greater plaque vulnerability.

## 1. Introduction

While significant progress has been made in cardiovascular disease management, ischemic heart disease persists as the predominant global mortality factor and continues to pose substantial public health challenges [1]. The rupture of vulnerable plaques and the subsequent acute thrombosis are considered the main mechanisms underlying most acute coronary syndromes and adverse cardiovascular events [2, 3, 4]. A study has shown that vulnerable plaque characteristics have higher predictive value for major adverse cardiovascular events (MACE) than the degree of arterial stenosis alone [5]. Therefore, early identification of vulnerable coronary plaques and proactive management of risk factors are extremely important in mitigating MACE. Vulnerable plaques typically exhibit a large lipid core, a thin fibrous cap, and infiltration by macrophages. Thin-cap fibroatheroma (TCFA) is the prototypical vulnerable plaque lesion and is widely recognized as a precursor to plaque rupture [6]. Intravascular optical coherence tomography (OCT), characterized by its high resolution and speed, can accurately identify vulnerable plaque features such as TCFA. It is currently the best intravascular imaging technique and is widely used in studies related to vulnerable plaques [7, 8].

Sleep disorders, including insufficient sleep or poor sleep quality, are increasingly recognized yet often overlooked determinants of health [9]. Sleep disorders can affect the functions of multiple systems, including the cardiovascular, respiratory, digestive, and nervous systems, through neural, humoral, and immune pathways and are direct causes or triggers of many fatal diseases [10]. Numerous studies have demonstrated that insufficient sleep and poor sleep quality are strongly associated with an increased prevalence of coronary heart disease (CHD) and MACE [11, 12, 13, 14, 15]. A healthy sleep pattern can significantly reduce the risk of CHD, cardiovascular disease, and stroke [16, 17]. In 2022, the American Heart Association updated its list of the top eight lifestyle habits that affect cardiovascular health. Sleep was included for the first time, with the statement that adequate sleep and good sleep quality are particularly important for maintaining cardiovascular health [18]. While extensive research has investigated the relationship between sleep duration, sleep quality, and cardiovascular diseases, no studies to date have explored their potential association with coronary artery plaque vulnerability.

We hypothesize that insufficient sleep or poor sleep quality may increase the risk of MACE by increasing the vulnerability of plaques. Therefore, this study aims to investigate the correlation between sleep duration, sleep quality, and vulnerability of coronary artery plaques in patients with stable angina.

## 2. Materials and Methods

### 2.1 Patient Population

A total of 260 patients with stable angina who underwent OCT in the Central China Fuwai Hospital of Zhengzhou University from September 2020 to September 2023 were included in this study. Patients who met the inclusion and exclusion criteria were asked to complete a questionnaire the day after the OCT examination. An experienced researcher provided detailed explanations of the questionnaire to the patients before they answered. The sleep duration of the patients was recorded according to the results of the questionnaire survey, and the sleep quality of the patients was evaluated by Pittsburgh sleep quality index (PSQI).

Inclusion criteria: (1) meet the American College of Cardiology/American Heart Association diagnostic criteria for stable angina [19]; (2) complete the coronary angiography (CAG) and OCT examination; (3) voluntarily sign informed consent and cooperate to complete the questionnaire; (4) age ≥30 years and ≤80 years.

Exclusion criteria: (1) patients with chronic occlusion, extreme tortuosity or severe calcification of vessels that cannot be examined by OCT; (2) patients with allergy to contrast media or contraindications to aspirin or ticagrelor who cannot undergo CAG; (3) patients with a history of coronary artery bypass grafting or coronary stent implantation; (4) patients with severe heart failure, endstage renal disease or severe liver dysfunction; (5) patients with other inflammation-related diseases such as cancer, infection and autoimmune diseases; (6) patients with severe mental illness or cognitive impairment who cannot cooperate to complete the questionnaire; (7) patients with poor OCT image quality that cannot be analyzed; (8) patients with missing clinical data.

Patients were divided into a TCFA group (86 cases) and a non-TCFA group (174 cases) according to whether there was at least one TCFA in the coronary artery on OCT examination. According to the optimal cut-off value of sleep duration in the receiver operating characteristic (ROC) curve analysis to predict the presence of TCFA, the patients were divided into a sleep duration ≤5.5 hours group (99 cases) and a sleep duration >5.5 hours group (161 cases). The patients were divided into a PSQI ≤9 group (173 cases) and a PSQI >9 group (87 cases) according to the optimal cut-off value of PSQI for predicting TCFA. This study was approved by the Medical Ethics Committee of the Central China Fuwai Hospital of Zhengzhou University. Informed consent was obtained from all patients and the data were kept confidential.

### 2.2 Clinical Data Collection

The clinical data of the patients were collected through the medical record system of the Central China Fuwai Hospital of Zhengzhou University. Data included: (1) General information: age, gender, body mass index, family history of CHD, history of diabetes, history of hypertension, history of hyperlipidemia, smoking history, and drinking history; (2) Laboratory examination indicators: red blood cell count, hemoglobin concentration, white blood cell count, platelet count, alanine transaminase, aspartate aminotransferase, total cholesterol (TC), triglyceride, low-density lipoprotein cholesterol (LDL-C), high-density lipoprotein cholesterol, glycosylated hemoglobin, albumin, globulin, urea, creatinine, uric acid, estimated glomerular filtration rate, fasting blood glucose, and C-reactive protein (CRP); (3) Results of ultrasound: left ventricular ejection fraction; (4) Medication history (regularly taken for more than one month): aspirin, statins, β-blockers, angiotensin-converting enzyme inhibitors/angiotensin receptor blockers, and alprazolam/estazolam.

### 2.3 Evaluation of Sleep Quality and Sleep Duration

All patients completed a questionnaire the day after undergoing OCT. Before administering the questionnaire, the contents of the questionnaire were explained in detail to the patients by trained research staff. The sleep duration survey was in the form of a self-report, with patients self-assessing the mean nighttime sleep duration, which was recorded by the investigator. PSQI was used to evaluate the sleep quality of patients in the past month, including 19 selfrating items and 5 evaluation items [20]. The PSQI consists of seven components: (1) subjective sleep quality, (2) sleep latency, (3) sleep persistence, (4) habitual sleep efficiency, (5) sleep disturbances, (6) daytime dysfunction, and (7) use of sleep medications. Each component was scored on a 0–3 scale, with a higher total PSQI indicating worse sleep quality.

### 2.4 OCT Imaging

All OCT images were obtained with Dragonfly OPTIS imaging catheter and intravascular catheter System (OPTIS™ MOBILE System; Abbott Vascular, Shanghai, China). Two experienced interventionalists completed the OCT examination of the target vessels by the non-balloon occlusion technique. The OCT imaging catheter was advanced to the distal vessel segment along the guidewire. Following contrast injection, automated pullback was initiated. Upon completion of image acquisition, the catheter was withdrawn. All OCT procedures were performed after coronary arteriography was completed, and intracoronary nitroglycerin was administered before the OCT examination.

### 2.5 OCT Analysis

**Fig. 1.**
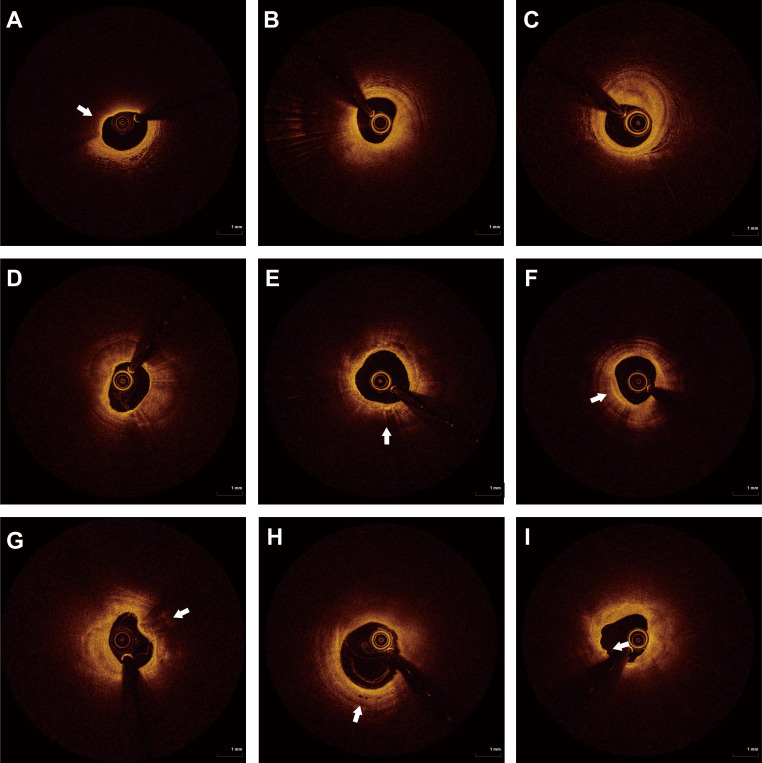
**OCT images of typical plaque features**. (A) TCFA. (B) Thick fiber cap lipid plaque. (C) Fibrous patches. (D) Calcified plaques. (E) Macrophage deposits. (F) Cholesterol crystals. (G) Calcified nodules. (H) Microchannel. (I) Plaque erosion. The white arrows point to typical plaque features on OCT images. OCT, optical coherence tomography; TCFA, Thin-cap fibroatheroma.

OCT images of all patients were analyzed at an independent laboratory (Henan Provincial Key Laboratory of Coronary Imaging Medicine, Zhengzhou, China) by two independent and experienced interventional technicians who were unaware of the patient’s clinical information. In case of inconsistent observations, concordant results were obtained from a third interventional technician. OCT images were analyzed according to consensus criteria [7]. The following parameters were analyzed: plaque length, proximal reference area, proximal reference diameter, distal reference area, distal reference diameter, minimum lumen area, thinnest fibrous cap thickness, maximal lipid pool arc, TCFA, macrophage deposits, cholesterol crystals, microchannels, calcified nodules, plaque erosion, and other relevant data. Three measurements were taken at the thinnest part of the fibrous cap, and the mean of the three measurements was calculated and recorded as the thinnest fibrous cap thickness. The lipid pool arc was measured at 1-mm intervals throughout the plaque, with the maximum value recorded as the maximal lipid pool arc. TCFA was defined as a lipid rich plaque exhibiting both: (1) maximal lipid pool arc ≥90°, and (2) fibrous cap thickness ≤65 µm [7, 21]. Typical OCT imaging features are shown in Fig. 1.

### 2.6 Statistical Analysis 

Data analysis was conducted with SPSS 25.0 (IBM Corp., Armonk, NY, USA). Normally distributed continuous variables were reported as mean ± SD and compared via independent *t*-tests, while non-normally distributed data were presented as median (interquartile range) and analyzed using the Mann-Whitney U test. For categorical data, frequencies (%) were calculated and group comparisons were performed using χ^2^ tests. The indicators with *p* < 0.05 in the univariate analysis were used as independent variables to conduct multivariate logistic regression analysis to determine the independent Predictors of TCFA. ROC curve analysis was conducted to assess the diagnostic performance of sleep duration and the PSQI in predicting TCFA. DeLong’s test was used to compare the areas under the curve (AUC) between these two predictors. The Spearman correlation analysis was used to evaluate the correlation of sleep duration and PSQI with the thinnest fibrous cap thickness and the maximum radian of the lipid pool in patients with stable angina. A significance level of *p* < 0.05 was considered statistically significant.

## 3. Results

### 3.1 Comparison of Clinical Data Between the TCFA Group and Non-TCFA Group

A total of 260 patients with stable angina who underwent OCT examination in the Central China Fuwai Hospital of Zhengzhou University from September 2020 to September 2023 were included. Patients were divided into a non-TCFA group (n = 174) and a TCFA (n = 86) group based on the presence of TCFA. Table 1 compares the clinical data of the two groups. The results of the univariate analysis showed that the sleep duration in the TCFA group was significantly shorter (5.51 ± 1.60 vs. 6.69 ± 1.59, *p* < 0.001). The PSQI in the TCFA group was significantly higher (9.94 ± 3.31 vs. 7.28 ± 2.95, *p* < 0.001). The prevalence of diabetes in the TCFA group was significantly higher [31 (36.05%) vs. 42 (24.14%), *p* < 0.05]. The levels of TC (3.70 ± 1.01 vs. 3.39 ± 0.85, *p* < 0.05), LDL-C (2.45 ± 0.85 vs. 2.08 ± 0.65, *p* < 0.05) and CRP (2.95 ± 0.94 vs. 2.56 ± 0.85, *p* < 0.05) in the TCFA group were significantly higher. There were no significant differences in other clinical data (all *p* > 0.05).

**Table 1.** **Comparison of clinical data between TCFA group and non-TCFA group**.

Variable		TCFA (n = 86)	non-TCFA (n = 174)	Test values	*p*-value
Age, years		58.26 ± 12.50	57.03 ± 11.05	0.770	0.443
Male, n (%)		68 (79.07)	138 (79.31)	0.002	0.964
BMI, kg/m^2^		26.08 ± 3.56	25.80 ± 2.70	0.687	0.493
Family history, n (%)		10 (11.63)	17 (9.77)	0.213	0.644
Diabetes, n (%)		31 (36.05)	42 (24.14)	4.042	0.044
Hypertension, n (%)		47 (54.65)	95 (54.60)	0.001	0.994
Hyperlipidemia, n (%)		19 (22.09)	33 (18.97)	0.352	0.553
Smoking history, n (%)		42 (48.84)	85 (48.85)	0.001	0.998
Drinking history, n (%)		21 (24.42)	60 (34.48)	2.718	0.099
RBC, 10^9^/L		4.41 ± 0.47	4.42 ± 0.52	–0.206	0.837
Hb, g/L		137.11 ± 12.76	137.43 ± 14.26	–0.173	0.863
WBC,10^9^/L		6.64 ± 1.47	6.56 ± 1.44	0.430	0.668
PLT, 10^6^/L		219.72 ± 54.11	217.50 ± 54.10	0.312	0.756
ALT, U/L		26.41 ± 10.10	27.42 ± 11.47	–0.692	0.489
AST, U/L		20.20 (16.00–25.85)	20.10 (15.60–25.78)	–0.202	0.840
TC, mmol/L		3.70 ± 1.01	3.39 ± 0.85	2.607	0.010
TG, mmol/L		1.48 (1.09–2.02)	1.32 (0.95–1.82)	–1.431	0.152
LDL-C, mmol/L		2.45 ± 0.85	2.08 ± 0.65	3.563	0.001
HDL-C, mmol/L		1.01 ± 0.18	1.02 ± 0.26	–0.252	0.802
HbA1c, %		5.84 (5.47–6.15)	5.75 (5.42–6.23)	–0.594	0.552
Albumin, g/L		41.39 ± 3.74	41.89 ± 3.48	–1.052	0.294
Globulin, g/L		22.03 ± 2.73	21.88 ± 3.45	0.380	0.704
BUN, mmol/L		5.28 ± 1.20	5.52 ± 1.43	–1.290	0.198
CR, µmol/L		72.08 ± 15.21	71.82 ± 13.83	0.141	0.888
UA, µmol/L		312.62 ± 75.67	317.71 ± 79.08	0.185	0.853
eGFR, mL/min/1.73 m^2^		91.06 ± 15.93	90.09 ± 13.40	0.516	0.606
Glucose, mmol/L		5.21 (4.61–6.23)	5.01 (4.54–5.92)	–0.212	0.832
CRP, mg/L		2.95 ± 0.94	2.56 ± 0.85	3.449	0.001
LVEF, %		57.87 ± 5.54	57.20 ± 5.70	0.901	0.368
Sleep duration, h		5.51 ± 1.60	6.69 ± 1.59	–5.623	<0.001
PSQI		9.94 ± 3.31	7.28 ± 2.95	6.573	<0.001
Medication					
	Aspirin, n (%)	48 (57.14)	118 (67.82)	3.592	0.058
	Statin, n (%)	50 (59.52)	120 (68.97)	2.980	0.084
	Beta blocker, n (%)	31 (36.05)	65 (37.36)	0.042	0.837
	ACEI/ARB, n (%)	25 (29.07)	39 (22.41)	1.374	0.241
	Alprazolam/Estazolam, n (%)	14 (16.28)	19 (10.92)	1.492	0.222

BMI, body mass index; RBC, red blood cells; Hb, hemoglobin; WBC, white blood cells; PLT, platelets; ALT, alanine transaminase; AST, aspartate aminotransferase; TC, total cholesterol; TG, triglycerides; LDL-C, low-density lipoprotein cholesterol; HDL-C, high-density lipoprotein cholesterol; HbA1c, glycated hemoglobin; BUN, blood urea nitrogen; CR, creatinine; UA, uric acid; eGFR, estimated glomerular filtration rate; CRP, C-reactive protein; LVEF, left ventricular ejection fraction; PSQI, Pittsburgh Sleep Quality Index; ACEI, angiotensin-converting enzyme inhibitor; ARB, angiotensin receptor blocker; TCFA, thin-cap fibroatheroma.

### 3.2 Multivariate Logistic Regression Analysis of TCFA

TCFA presence (non-TCFA group assigned a value of 0, TCFA group assigned a value of 1) served as the dependent variable. All variables demonstrating statistical significance in univariate analysis were entered into a multivariate logistic regression model to identify independent predictors of TCFA among stable angina patients. The results are detailed in Table 2. Sleep duration, PSQI, history of diabetes, LDL-C, and CRP were identified as independent predictors of TCFA in patients with stable angina [Sleep duration: OR = 0.764, 95% CI (0.625–0.932); PSQI: OR = 1.233, 95% CI (1.108–1.372); diabetes: OR = 2.081, 95% CI (1.078–4.015); LDL-C: OR = 2.222, 95% CI (1.109–4.453); CRP: OR = 1.543, 95% CI (1.097–2.172)].

**Table 2.** **Multivariate logistic regression analysis of TCFA**.

Variable	β	Wald	OR	95% CI	*p*-value
Diabetes, n (%)	–0.733	4.773	2.081	1.078–4.015	0.029
TC, mmol/L	–0.135	0.221	0.874	0.498–1.534	0.638
LDL-C, mmol/L	0.798	5.065	2.222	1.109–4.453	0.024
CRP, mg/L	0.434	6.206	1.543	1.097–2.172	0.013
Sleep duration, h	–0.270	7.031	0.764	0.625–0.932	0.008
PSQI	0.209	14.743	1.233	1.108–1.372	<0.001
Constant	–3.586	8.443	0.028	-	0.004

TC, total cholesterol; LDL-C, low-density lipoprotein cholesterol; CRP, C-reactive protein; PSQI, Pittsburgh Sleep Quality Index; β, beta; OR, odds ratio; CI, confidence interval; TCFA, thin-cap fibroatheroma.

### 3.3 Value of Sleep Duration and Quality in Predicting TCFA

**Fig. 2.**
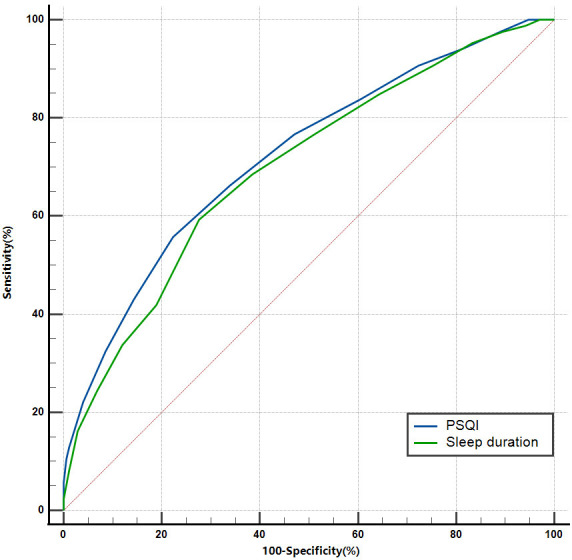
**ROC curve of sleep duration and PSQI for predicting the presence of TCFA in patients with stable angina**. PSQI, Pittsburgh Sleep Quality Index; TCFA, thin-cap fibroatheroma; ROC, receiver operating characteristic.

The ROC curve analysis further validated the predictive value of sleep duration and PSQI for the presence of TCFA in patients with stable angina. The results are shown in Fig. 2.

The AUC for sleep duration predicting TCFA was 0.698 (95% CI: 0.639–0.754; *p* < 0.001), with an optimal cutoff value of 5.5 (0.3172), corresponding to a sensitivity of 59.30% and specificity of 72.41%.

The AUC for PSQI predicting TCFA was 0.721 (95% CI: 0.663–0.775; *p* < 0.001), with an optimal cutoff value of 9 (0.3340), corresponding to a sensitivity of 55.81% and specificity of 77.59%.

Although the area under the curve of PSQI predicting TCFA was larger than that of sleep duration, there was no statistical difference (*p* = 0.573).

### 3.4 Comparison of Baseline Clinical and OCT Characteristics According to the Cut-Off Value of Sleep Duration

Patients were divided into two groups according to the optimal cut-off value of sleep time: sleep duration >5.5 hours group (n = 161) and sleep duration ≤5.5 hours group (n = 99). The results of the comparison of baseline clinical and OCT characteristics between the two groups are shown in Table 3. Compared with patients with sleep duration >5.5 hours, those with sleep duration ≤5.5 hours had higher levels of LDL [2.25 (1.83–2.79) vs. 2.03 (1.69–2.50), *p* < 0.05] and CRP [2.946 ± 0.942 vs. 2.527 ± 0.829, *p* < 0.001]. The thinnest fibrous cap in patients with sleep duration ≤5.5 was thinner than in those with sleeping duration >5.5 hours [65.00 (44.00–136.00) vs. 99.00 (72.50–199.00), *p* < 0.001]. Patients with sleep duration ≤5.5 hours had a larger curvature of maximum lipid pool [220.00 (180.00–300.00) vs. 180.00 (150.00–230.00), *p* < 0.001], and a smaller minimum lumen area (2.16 ± 0.50 vs. 2.45 ± 0.83, *p* < 0.05). Patients with sleep duration ≤5.5 hours had significantly higher rates of TCFA [51 (51.52) vs. 35 (21.74), *p* < 0.001] and macrophage deposition [26 (26.26) vs. 22 (13.66), *p* < 0.05] than those with sleep duration >5.5 hours. Patients with shorter sleep duration had more characteristics of plaque vulnerability.

**Table 3.** **Comparison of baseline clinical and OCT characteristics according to the cut-off value of sleep duration**.

Variable		Sleep duration ≤5.5 h (n = 99)	Sleep duration >5.5 h (n = 161)	Test values	*p*-value
Age, years		57.60 ± 11.39	57.36 ± 12.38	0.154	0.878
Male, n (%)		80 (80.81)	126 (78.26)	0.242	0.623
BMI, kg/m^2^		25.80 (23.60–27.70)	25.40 (24.20–27.35)	–0.053	0.958
Family history, n (%)		11 (11.11)	16 (9.94)	0.091	0.763
Diabetes, n (%)		28 (28.28)	45 (27.95)	0.003	0.954
Hypertension, n (%)		60 (60.61)	82 (50.93)	2.315	0.128
Hyperlipidemia, n (%)		25 (25.25)	27 (16.77)	2.757	0.097
Smoking history, n (%)		50 (50.51)	77 (47.83)	0.176	0.675
Drinking history, n (%)		26 (26.26)	54 (33.54)	1.524	0.217
TC, mmol/L		3.586 ± 0.943	3.431 ± 0.891	1.336	0.183
TG, mmol/L		1.37 (1.04–1.83)	1.32 (0.95–1.91)	–0.034	0.973
LDL-C, mmol/L		2.25 (1.83–2.79)	2.03 (1.69–2.50)	–2.377	0.017
HDL-C, mmol/L		0.98 (0.86–1.12)	1.01 (0.85–1.20)	–1.198	0.231
HbA1c, %		5.73 (5.46–6.14)	5.85 (5.41–6.23)	–0.468	0.640
Glucose, mmol/L		4.98 (4.44–5.86)	5.15 (4.57–6.16)	–1.351	0.177
CRP, mg/L		2.946 ± 0.942	2.527 ± 0.829	3.754	<0.001
Target vessels, n (%)				3.225	0.199
	LAD	64 (64.65)	120 (74.53)		
	LCX	15 (15.15)	15 (9.32)		
	RCA	20 (20.20)	26 (16.15)		
	Plaque length, mm	22.71 ± 7.57	21.75 ± 7.95	0.966	0.335
Proximal reference area, mm^2^		9.57 ± 2.63	10.04 ± 3.38	–1.176	0.241
Proximal reference diameter, mm		3.47 ± 0.50	3.52 ± 0.61	–0.669	0.485
Distal reference area, mm^2^		7.64 ± 2.38	8.10 ± 2.44	–1.469	0.143
Distal reference diameter, mm		3.07 ± 0.49	3.15 ± 0.50	–1.240	0.216
Minimum lumen area, mm^2^		2.36 ± 0.50	2.65 ± 0.83	–2.752	0.006
Thinnest fibrous cap thickness, µm		65.00 (44.00–136.00)	99.00 (72.50–199.00)	–4.546	<0.001
Maximal lipid pool arc, deg		220.00 (180.00–300.00)	180.00 (150.00–230.00)	–4.230	<0.001
TCFA, n (%)		51 (51.52)	35 (21.74)	24.554	<0.001
Macrophage, n (%)		26 (26.26)	22 (13.66)	6.463	0.011
Cholesterol crystal, n (%)		53 (53.54)	82 (50.93)	0.166	0.683
Microchannel, n (%)		21 (21.21)	50 (31.06)	2.993	0.084
Calcified nodule, n (%)		6 (6.06)	12 (7.45)	0.185	0.667
Plaque erosion, n (%)		21 (21.21)	20 (12.42)	3.566	0.059

BMI, body mass index; TC, total cholesterol; TG, triglycerides; LDL-C, low-density lipoprotein cholesterol; HDL-C, high-density lipoprotein cholesterol; HbA1c, glycated hemoglobin; CRP, C-reactive protein; LAD, left anterior descending artery; LCX, left circumflex artery; RCA, right coronary artery; TCFA, thin-cap fibroatheroma; OCT, optical coherence tomography.

### 3.5 Comparison of Baseline Clinical and OCT Characteristics According to the Cut-Off Value of PSQI

Patients were divided into two groups according to the optimal cut-off value of PSQI: PSQI ≤9 group (n = 173) and PSQI >9 group (n = 87). The results of the comparison of baseline clinical and OCT imaging characteristics between the two groups are shown in Table 4. Compared with patients with PSQI ≤9, patients with PSQI >9 had a higher CRP level [2.60 ± 0.83 vs. 2.86 ± 1.00, *p* < 0.05]. The thinnest fibrous cap in patients with PSQI >9 was thinner than in those with PSQI ≤9 [62.00 (41.00–135.00) vs. 99.00 (72.00–201.50), *p* < 0.001]. Patients with PSQI >9 had a larger maximal lipid pool arc [240.00 (180.00–300.00) vs. 180.00 (150.00–220.00), *p* < 0.001], and a smaller minimum lumen area (2.19 ± 0.65 vs. 2.41 ± 0.91, *p* < 0.05). The incidence of TCFA [48 (55.17) vs. 38 (21.96), *p* < 0.001], macrophage deposition [22 (25.29) vs. 26 (15.03), *p* < 0.05], and plaque erosion [22 (25.29) vs. 19 (10.98), *p* < 0.05] in patients with PSQI >9 was significantly higher. Patients with poor sleep quality had more characteristics of plaque vulnerability.

**Table 4.** **Comparison of baseline clinical and OCT characteristics according to the cut-off value of PSQI**.

Variable		PSQI ≤9 (n = 173)	PSQI >9 (n = 87)	Test values	*p*-value
Age, years		56.79 ± 12.24	58.77 ± 11.43	1.260	0.209
Male, n (%)		143 (82.66)	63 (72.41)	3.692	0.055
BMI, kg/m^2^		25.40 (24.25–27.45)	25.50 (23.80–27.80)	–0.213	0.831
Family history, n (%)		21 (12.14)	6 (6.90)	1.709	0.191
Diabetes, n (%)		47 (27.17)	26 (29.89)	0.212	0.645
Hypertension, n (%)		93 (53.76)	49 (56.32)	0.154	0.695
Hyperlipidemia, n (%)		31 (17.92)	21 (24.14)	1.399	0.237
Smoking history, n (%)		88 (50.87)	39 (44.83)	0.845	0.358
Drinking history, n (%)		60 (34.68)	20 (22.99)	3.716	0.054
TC, mmol/L		3.38 (2.81–3.93)	3.45 (2.84–4.21)	–0.548	0.584
TG, mmol/L		1.38 (0.95–1.88)	1.27 (1.08–1.85)	–0.54	0.957
LDL-C, mmol/L		2.08 (1.70–2.52)	2.21 (1.78–2.62)	–1.228	0.220
HDL-C, mmol/L		0.99 (0.84–1.17)	1.01 (0.88–1.16)	–0.843	0.399
HbA1c, %		5.79 (5.43–6.21)	5.75 (5.43–6.18)	–0.032	0.974
Glucose, mmol/L		5.08 (4.59–5.98)	4.98 (4.43–5.92)	–1.018	0.309
CRP, mg/L		2.60 ± 0.83	2.86 ± 1.00	2.178	0.030
Target vessels, n (%)				0.313	0.855
	LAD	124 (71.68)	60 (68.97)		
	LCX	20 (11.56)	10 (11.49)		
	RCA	26 (15.03)	17 (19.54)		
Plaque length, mm		21.93 ± 7.48	22.50 ± 8.45	0.553	0.581
Proximal reference area, mm^2^		9.62 ± 3.22	10.22 ± 2.67	1.510	0.132
Proximal reference diameter, mm		3.44 ± 0.59	3.58 ± 0.49	1.923	0.056
Distal reference area, mm^2^		7.95 ± 2.49	7.87 ± 2.28	–0.236	0.813
Distal reference diameter, mm		3.13 ± 0.51	3.12 ± 0.46	–0.136	0.892
Minimum lumen area, mm^2^		2.61 ± 0.91	2.39 ± 0.65	–2.178	0.030
Thinnest fibrous cap thickness, µm		99.00 (72.00–201.50)	62.00 (41.00–135.00)	–5.011	<0.001
Maximal lipid pool arc, deg		180.00 (150.00–220.00)	240.00 (180.00–300.00)	–5.134	<0.001
TCFA, n (%)		38 (21.96)	48 (55.17)	28.837	<0.001
Macrophage, n (%)		26 (15.03)	22 (25.29)	4.047	0.044
Cholesterol crystal, n (%)		86 (49.71)	49 (56.32)	1.013	0.314
Microchannel, n (%)		52 (30.06)	19 (21.84)	1.970	0.160
Calcified nodule, n (%)		12 (6.94)	6 (6.90)	0.001	0.990
Plaque erosion, n (%)		19 (10.98)	22 (25.29)	8.918	0.003

BMI, body mass index; TC, total cholesterol; TG, triglycerides; LDL-C, low-density lipoprotein cholesterol; HDL-C, high-density lipoprotein cholesterol; HbA1c, glycated hemoglobin; CRP, C-reactive protein; LAD, left anterior descending artery; LCX, left circumflex artery; RCA, right coronary artery; TCFA, thin-cap fibroatheroma; PSQI, Pittsburgh Sleep Quality Index; OCT, optical coherence tomography.

### 3.6 Spearman Correlation Analysis

**Fig. 3.**
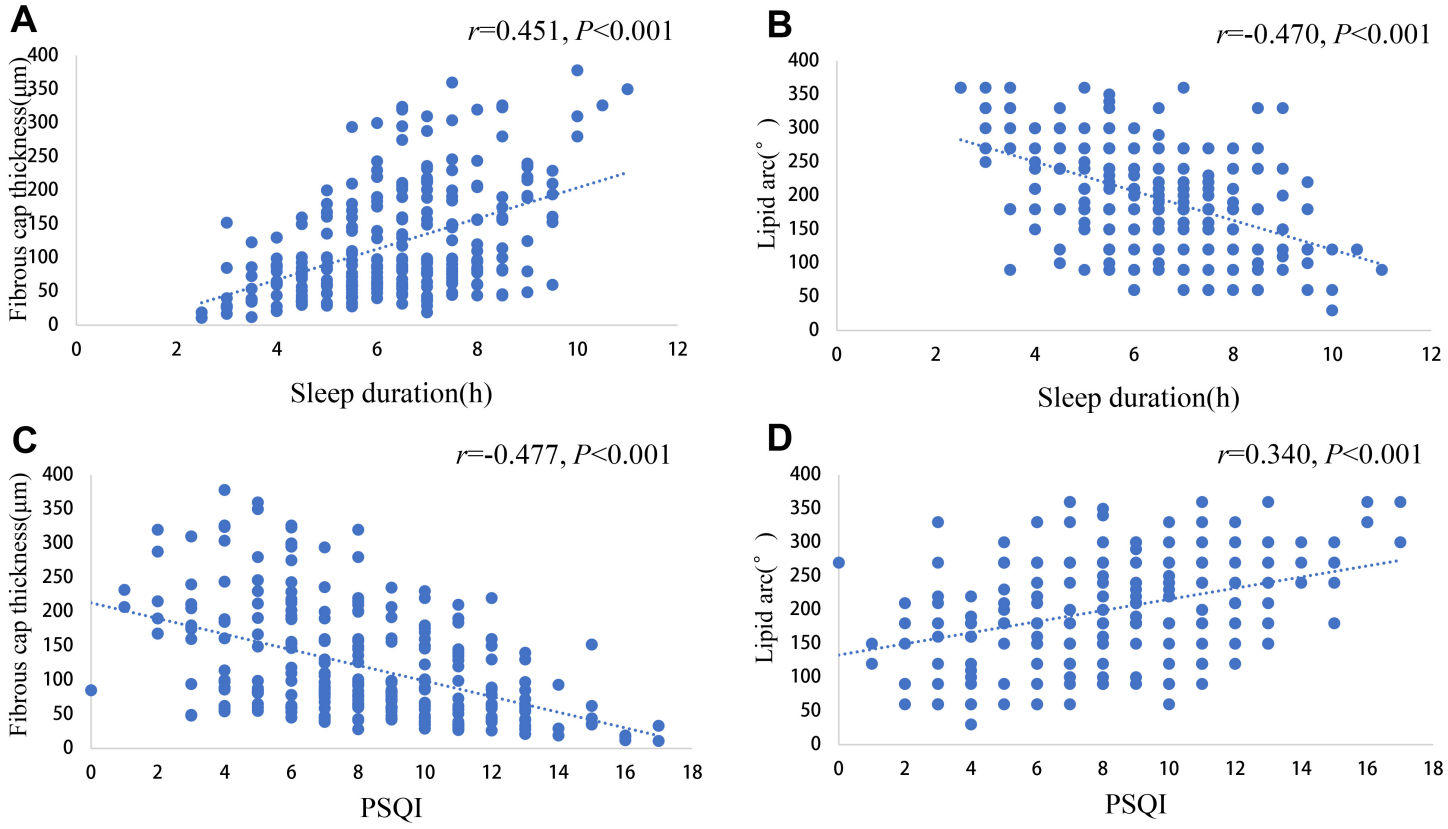
**Scatter plot of correlations of sleep duration and PSQI with thinnest fibrous cap thickness and maximal radian of lipid pool**. (A) Scatter plot showing the correlation between sleep duration and the thinnest cap thickness. (B) Scatter plot showing the correlation between sleep duration and the radian of maximum lipid pool. (C) Scatter plot showing the correlation between PSQI and the thinnest fiber cap thickness. (D) Scatter plot showing the correlation between PSQI and the radian of maximum lipid pool. PSQI, Pittsburgh Sleep Quality Index.

Spearman correlation analysis was used to evaluate the correlation of sleep duration and PSQI with the thinnest fibrous cap thickness and the maximum radian of lipid pool. The results are shown in Fig. 3. Sleep duration was positively correlated with the thinnest cap thickness (r = 0.451; *p* < 0.001), and negatively correlated with the radian of the maximum lipid pool (r = –0.470; *p* < 0.001). PSQI was negatively correlated with the thinnest fiber cap thickness (r = –0.477; *p* < 0.001), and positively correlated with the radian of the maximum lipid pool (r = 0.340; *p* < 0.001). Patients with shorter sleep duration had thinner fibrous caps and a larger lipid pool arc. Similarly, those with poorer sleep quality also showed thinner fibrous caps and a larger lipid arc.

## 4. Discussion

This study explored the association of sleep duration and quality with coronary atherosclerotic plaque vulnerability. The analysis demonstrated that sleep duration and quality were closely related to the presence of TCFA in patients with stable angina. Patients in the TCFA group had shorter sleep duration and worse sleep quality. Both sleep duration and quality are independent predictors of TCFA in patients with stable angina. Stable angina patients with sleep duration ≤5.5 hours exhibit more features of plaque vulnerability. Specifically, shorter sleep duration is associated with thinner fibrous cap thickness, larger lipid pool arc, and higher plaque vulnerability. Similarly, stable angina patients with PSQI >9 show greater signs of vulnerable plaques. Poorer sleep quality correlates with thinner fibrous caps, larger lipid pool arcs, and increased plaque instability.

### 4.1 Vulnerable Plaque of Coronary Atherosclerosis and Common Influencing Factors

The formation and development of coronary atherosclerotic plaques is a dynamic and evolving process. Vulnerable plaques, which are prone to rupture and thrombosis, play a significant role in the rapid progression of the disease. These plaques typically have a thinner fibrous cap, a larger lipid necrotic core, and exhibit features of vulnerability such as macrophage infiltration, calcified nodules, plaque erosion, cholesterol crystals and microchannels [22]. The rupture of vulnerable plaques and subsequent acute thrombosis are major causes of the acute coronary syndrome and the occurrence of MACE [23, 24]. Research has shown that TCFA are independent predictors of cardiac death, non-culprit lesion-related myocardial infarction (MI), and composite endpoints involving coronary revascularization during follow-up after MI [25]. Further evidence confirms that TCFA serves as the most potent independent predictor of composite endpoints, encompassing cardiac death, target vessel MI, target lesion revascularization, and unstable angina hospitalization in patients with stable angina [26]. TCFA represents the hallmark morphological feature of vulnerable plaques, demonstrating a robust association with MACE [27].

OCT enables the quantitative analysis of fibrous cap thickness and lipid pool curvature, making it the most accurate technique for evaluating TCFA [7, 8]. In this study, OCT revealed that approximately one-third of patients with stable angina exhibited TCFA, suggesting that nearly one-third of stable angina patients are at risk of rapidly progressing to the acute coronary syndrome. The study by Nasu further showed that in untreated patients with stable angina, the incidence of TCFA was as high as 53% [28]. Therefore, timely detection and active treatment of TCFA in stable angina patients to reduce the risk of plaque rupture and acute thrombosis is crucial for preventing MACE and improving clinical outcomes. Notably, more than half of the stable angina patients in this study had received lipid-lowering therapy for over a month, which may explain the relatively low incidence of TCFA.

The analysis further revealed a significantly higher diabetes prevalence among TCFA group patients compared to non-TCFA group patients. Meanwhile, the TCFA group had significantly higher levels of TC, LDL-C, and CRP. This is consistent with previous studies showing that diabetes, LDL-C, and CRP are independent predictors of the presence of TCFA in CHD patients [22, 28, 29, 30]. Blood glucose levels, lipid profiles, and inflammatory status are key factors affecting vulnerable plaques and the formation of TCFA. OCT results showed that coronary atherosclerotic plaques in patients with diabetes or prediabetes were more diffusely distributed and exhibited higher vulnerability [30]. Multivariate analysis revealed that altered glucose metabolism was an independent predictor of TCFA and plaque erosion. Research by Nasu demonstrated that LDL-C, CRP, and the ratio of campesterol to cholesterol were positively correlated with fibrous cap thickness and negatively correlated with the volume of the lipid necrotic core, and all were independent predictors of TCFA [28]. Additionally, Kini also found that lipid-lowering therapy significantly increased fibrous cap thickness and reduced the incidence of TCFA [31]. In this study, the proportion of patients on statin therapy was lower in the TCFA group, although this difference was not statistically significant.

### 4.2 Association of Sleep Duration and Quality With Plaque Vulnerability

Increasing evidence suggests that sleep is an important factor influencing cardiovascular diseases. Poor sleep quality and insufficient sleep are both associated with an increased incidence of CHD [32]. Less than 5 hours of sleep is closely related to the occurrence of unstable angina pectoris and MI [33]. A meta analysis revealed that compared to 7 hours of sleep per day, each hour of reduced sleep duration was associated with a 6%, 6%, 7%, and 5% increase in all cause mortality, total cardiovascular disease risk, CHD risk, and stroke risk [12]. In addition, an 8-year follow-up study of 9570 patients showed that after adjusting for conventional CHD risk factors and sleep time, individuals with poor sleep quality had a significantly increased relative risk of developing CHD [34]. Yang *et al*. [35] demonstrated that impaired sleep quality independently predicts complex coronary lesions and elevates the risk of MACE. Nevertheless, the relationship between sleep duration, sleep quality and the vulnerability of atherosclerotic plaques remains unclear.

The results showed that all patients in this study had an average sleep duration of less than 7 hours, and an average PSQI greater than 7, indicating that patients with stable angina generally have poor sleep. Furthermore, the sleep duration in the TCFA group was shorter and the sleep quality was worse. Multivariate logistic regression analysis demonstrated that sleep duration and quality were independent predictors of TCFA. This suggests that patients with severe sleep deprivation and poor sleep quality have more vulnerable plaques. These findings provide theoretical support for the increased incidence of MACE in patients with insufficient sleep and poor sleep quality [15, 33, 36]. Additionally, ROC curve analysis further indicated that sleep duration and quality have predictive value for the presence of TCFA in stable angina patients. Some studies have suggested that sleep quality may exert a more pronounced influence on CHD than sleep duration [37, 38]. The PSQI evaluates sleep quality across seven distinct domains, providing a comprehensive assessment of overall sleep status [39]. However, although PSQI-assessed sleep quality yielded a larger AUC for TCFA prediction compared to sleep duration, no statistically significant difference was observed between the two predictors. This lack of significance may be attributable to the relatively small sample size in the present study.

Further analysis demonstrated that patients with sleep duration ≤5.5 hours or PSQI >9 showed significantly higher incidence of TCFA and macrophage deposition, along with thinner fibrous caps, larger lipid pool radians, and smaller minimal lumen areas. Notably, shorter sleep duration and poorer sleep quality were strongly correlated with progressive thinning of fibrous caps, expansion of lipid pool curvature, and elevated plaque vulnerability, suggesting a dose-dependent relationship between sleep deprivation and atherosclerotic plaque instability. A study investigating the relationship between coronary artery plaque characteristics and clinical events within one year showed that minimal lumen area, fibrous cap thickness, lipid pool curvature, and macrophage deposition were associated with an increased risk of MI and cardiac death as composite endpoints [40]. These findings also explain why patients with poor sleep have a higher incidence of MACE. However, the relationship between prolonged sleep duration and CHD remains clinically contentious. Current evidence demonstrates considerable inconsistency, with multiple studies failing to establish a significant association [41, 42]. In our study, there were few patients with sleep durations exceeding 9 hours, and no effect of prolonged sleep duration on plaque vulnerability was observed.

### 4.3 Potential Mechanisms of Insufficient Sleep and Poor Sleep Quality Associated With Plaque Vulnerability

The exact etiology and mechanisms behind the formation of vulnerable plaques remain unclear. Previous studies have shown that chronic inflammation, oxidative stress, endothelial cell damage, dysregulation of glucose and lipid metabolism, and hemodynamic changes are closely associated with the development of vulnerable plaques [22, 29].

Studies have shown that insufficient sleep and poor sleep quality can increase the levels of inflammatory cells and inflammatory factors, and trigger a chronic inflammatory state [43, 44, 45]. These inflammatory responses can activate monocyte-derived macrophages, which secrete matrix metalloproteinases (MMPs), capable of degrading collagen in the fibrous cap, thereby increasing the vulnerability of plaques [46, 47]. The results of this study found that the CRP level in the TCFA group was significantly higher. CRP, as a marker of chronic inflammation, is closely related to the vulnerability of plaques. The study also found that the CRP level in patients with poor sleep quality or insufficient sleep was significantly higher. These findings suggest that inflammatory pathways likely mediate the observed association between poor sleep and enhanced plaque vulnerability.

There are also studies showing that poor sleep can lead to the accumulation of reactive oxygen species and an increase in the levels of oxidative stress [48, 49, 50]. Additionally, short-term sleep restriction is associated with an increase in the level of myeloperoxidase (MPO) [51]. MPO can increase the content of oxidized LDL-C and promote the expression of MMPs, thereby promoting the accumulation of lipid in plaques and increasing the vulnerability of plaques [52, 53]. Therefore, the oxidative stress level may also be one of the potential mechanisms by which insufficient sleep and poor sleep quality increase the vulnerability of plaques, but this needs to be further verified.

Sleep is closely related to various risk factors for cardiovascular diseases. Insufficient sleep, poor sleep quality, and sleep-related diseases increase the risks of hypertension, diabetes, hyperlipidemia, smoking, and mental disorders (such as anxiety and depression) [54, 55, 56, 57, 58]. Studies have shown that most risk factors for cardiovascular diseases are closely related to the vulnerability of plaques, and some are even independent risk factors for vulnerable plaques [59, 60, 61]. This study found that more patients in the TCFA group had abnormal blood sugar and lipid levels. Patients with insufficient sleep had significantly higher levels of LDL. Although patients with insufficient sleep and poor sleep quality had a higher prevalence of hypertension, diabetes, and hyperlipidemia, there was no statistical difference. This discrepancy likely reflects the limited sample size. Therefore, although these risk factors can partially explain the association between insufficient sleep, poor sleep quality, and the vulnerability of plaques, the specific mechanism still needs to be defined.

### 4.4 Study Limitations

Several limitations should be acknowledged in this study. First, as a single-center study with a limited sample size, our research may not have adequately controlled for all potential confounding variables. Future multi-center studies with larger cohorts are warranted to validate these findings. Second, this study used a questionnaire to assess sleep status, which is highly subjective and affects the accuracy of the results. Third, the limited sample size of long duration sleepers in our cohort precluded conclusive assessment of its association with plaque vulnerability. Finally, although we demonstrated that insufficient sleep and poor sleep quality could induce the formation of coronary vulnerable plaques, the precise pathophysiological mechanisms underlying these observations require clarification through additional mechanistic studies.

## 5. Conclusion

Our study demonstrates that both sleep duration and sleep quality are independently associated with plaque vulnerability in patients with stable angina. Patients with either short sleep duration or poor sleep quality showed significantly higher plaque vulnerability. These sleep parameters exhibit predictive value for assessing plaque vulnerability. These findings provide novel clinical insights regarding the relationship between sleep patterns and MACE.

## Data Availability

The datasets utilized or analyzed during the current study are available from the corresponding author upon reasonable request.

## Abbreviations

OCT, optical coherence tomography; TCFA, thin-cap fibroatheroma; MACE, major adverse cardiovascular events; CHD, coronary heart disease; MI, myocardial infarction; CAG, coronary angiography; PSQI, Pittsburgh sleep quality index; TC, total cholesterol; LDL-C, low-density lipoprotein cholesterol; CRP, C-reactive protein; MMPs, matrix metalloproteinases.

## References

[1] Roth GA, Mensah GA, Johnson CO, Addolorato G, Ammirati E, Baddour LM (2020). Global Burden of Cardiovascular Diseases and Risk Factors, 1990-2019: Update From the GBD 2019 Study. *Journal of the American College of Cardiology*.

[2] Falk E, Shah PK, Fuster V (1995). Coronary plaque disruption. *Circulation*.

[3] Mushenkova NV, Summerhill VI, Zhang D, Romanenko EB, Grechko AV, Orekhov AN (2020). Current Advances in the Diagnostic Imaging of Atherosclerosis: Insights into the Pathophysiology of Vulnerable Plaque. *International Journal of Molecular Sciences*.

[4] Gaba P, Gersh BJ, Muller J, Narula J, Stone GW (2023). Evolving concepts of the vulnerable atherosclerotic plaque and the vulnerable patient: implications for patient care and future research. *Nature Reviews. Cardiology*.

[5] Stone GW, Maehara A, Lansky AJ, de Bruyne B, Cristea E, Mintz GS (2011). A prospective natural-history study of coronary atherosclerosis. *The New England Journal of Medicine*.

[6] Kinoshita D, Suzuki K, Fujimoto D, Niida T, Minami Y, Dey D (2025). High-risk plaque features and perivascular inflammation. *Journal of Cardiovascular Computed Tomography*.

[7] Tearney GJ, Regar E, Akasaka T, Adriaenssens T, Barlis P, Bezerra HG (2012). Consensus standards for acquisition, measurement, and reporting of intravascular optical coherence tomography studies: a report from the International Working Group for Intravascular Optical Coherence Tomography Standardization and Validation. *Journal of the American College of Cardiology*.

[8] Nakahara T, Strauss HW, Narula J, Jinzaki M (2023). Vulnerable Plaque Imaging. *Seminars in Nuclear Medicine*.

[9] Consensus Conference Panel, Watson NF, Badr MS, Belenky G, Bliwise DL, Buxton OM (2015). Joint Consensus Statement of the American Academy of Sleep Medicine and Sleep Research Society on the Recommended Amount of Sleep for a Healthy Adult: Methodology and Discussion. *Sleep*.

[10] Ferrie JE, Kumari M, Salo P, Singh-Manoux A, Kivimäki M (2011). Sleep epidemiology–a rapidly growing field. *International Journal of Epidemiology*.

[11] St-Onge MP, Grandner MA, Brown D, Conroy MB, Jean-Louis G, Coons M (2016). Sleep Duration and Quality: Impact on Lifestyle Behaviors and Cardiometabolic Health: A Scientific Statement From the American Heart Association. *Circulation*.

[12] Yin J, Jin X, Shan Z, Li S, Huang H, Li P (2017). Relationship of Sleep Duration With All-Cause Mortality and Cardiovascular Events: A Systematic Review and Dose-Response Meta-Analysis of Prospective Cohort Studies. *Journal of the American Heart Association*.

[13] Kwok CS, Kontopantelis E, Kuligowski G, Gray M, Muhyaldeen A, Gale CP (2018). Self-Reported Sleep Duration and Quality and Cardiovascular Disease and Mortality: A Dose-Response Meta-Analysis. *Journal of the American Heart Association*.

[14] Cappuccio FP, Cooper D, D’Elia L, Strazzullo P, Miller MA (2011). Sleep duration predicts cardiovascular outcomes: a systematic review and meta-analysis of prospective studies. *European Heart Journal*.

[15] Andrechuk CRS, Ceolim MF (2016). Sleep quality and adverse outcomes for patients with acute myocardial infarction. *Journal of Clinical Nursing*.

[16] Fan M, Sun D, Zhou T, Heianza Y, Lv J, Li L (2020). Sleep patterns, genetic susceptibility, and incident cardiovascular disease: a prospective study of 385 292 UK biobank participants. *European Heart Journal*.

[17] Nambiema A, Lisan Q, Vaucher J, Perier MC, Boutouyrie P, Danchin N (2023). Healthy sleep score changes and incident cardiovascular disease in European prospective community-based cohorts. *European Heart Journal*.

[18] Lloyd-Jones DM, Allen NB, Anderson CAM, Black T, Brewer LC, Foraker RE (2022). Life’s Essential 8: Updating and Enhancing the American Heart Association’s Construct of Cardiovascular Health: A Presidential Advisory From the American Heart Association. *Circulation*.

[19] Fraker TD, Fihn SD, Gibbons RJ, 2002 Chronic Stable Angina Writing Committee, American College of Cardiology, American Heart Association (2007). 2007 chronic angina focused update of the ACC/AHA 2002 guidelines for the management of patients with chronic stable angina: a report of the American College of Cardiology/American Heart Association Task Force on Practice Guidelines Writing Group to develop the focused update of the 2002 guidelines for the management of patients with chronic stable angina. *Journal of the American College of Cardiology*.

[20] Madsen MT, Huang C, Zangger G, Zwisler ADO, Gögenur I (2019). Sleep Disturbances in Patients With Coronary Heart Disease: A Systematic Review. *Journal of Clinical Sleep Medicine: JCSM: Official Publication of the American Academy of Sleep Medicine*.

[21] Deng F, Li D, Lei L, Yang Q, Li Q, Wang H (2021). Association between apolipoprotein B/A1 ratio and coronary plaque vulnerability in patients with atherosclerotic cardiovascular disease: an intravascular optical coherence tomography study. *Cardiovascular Diabetology*.

[22] Stefanadis C, Antoniou CK, Tsiachris D, Pietri P (2017). Coronary Atherosclerotic Vulnerable Plaque: Current Perspectives. *Journal of the American Heart Association*.

[23] Erlinge D, Maehara A, Ben-Yehuda O, Bøtker HE, Maeng M, Kjøller-Hansen L (2021). Identification of vulnerable plaques and patients by intracoronary near-infrared spectroscopy and ultrasound (PROSPECT II): a prospective natural history study. *Lancet (London, England)*.

[24] Gallone G, Bellettini M, Gatti M, Tore D, Bruno F, Scudeler L (2023). Coronary Plaque Characteristics Associated With Major Adverse Cardiovascular Events in Atherosclerotic Patients and Lesions: A Systematic Review and Meta-Analysis. *JACC. Cardiovascular Imaging*.

[25] Jiang S, Fang C, Xu X, Xing L, Sun S, Peng C (2023). Identification of High-Risk Coronary Lesions by 3-Vessel Optical Coherence Tomography. *Journal of the American College of Cardiology*.

[26] Fabris E, Berta B, Roleder T, Hermanides RS, IJsselmuiden AJJ, Kauer F (2022). Thin-Cap Fibroatheroma Rather Than Any Lipid Plaques Increases the Risk of Cardiovascular Events in Diabetic Patients: Insights From the COMBINE OCT-FFR Trial. *Circulation. Cardiovascular Interventions*.

[27] Burke AP, Farb A, Malcom GT, Liang YH, Smialek J, Virmani R (1997). Coronary risk factors and plaque morphology in men with coronary disease who died suddenly. *The New England Journal of Medicine*.

[28] Nasu K, Terashima M, Habara M, Ko E, Ito T, Yokota D (2013). Impact of cholesterol metabolism on coronary plaque vulnerability of target vessels: a combined analysis of virtual histology intravascular ultrasound and optical coherence tomography. *JACC. Cardiovascular Interventions*.

[29] Bentzon JF, Otsuka F, Virmani R, Falk E (2014). Mechanisms of plaque formation and rupture. *Circulation Research*.

[30] De Rosa R, Vasa-Nicotera M, Leistner DM, Reis SM, Thome CE, Boeckel JN (2017). Coronary Atherosclerotic Plaque Characteristics and Cardiovascular Risk Factors - Insights From an Optical Coherence Tomography Study. *Circulation Journal: Official Journal of the Japanese Circulation Society*.

[31] Kini AS, Vengrenyuk Y, Shameer K, Maehara A, Purushothaman M, Yoshimura T (2017). Intracoronary Imaging, Cholesterol Efflux, and Transcriptomes After Intensive Statin Treatment: The YELLOW II Study. *Journal of the American College of Cardiology*.

[32] Zhang B, Wang Y, Liu X, Zhai Z, Sun J, Yang J (2021). The association of sleep quality and night sleep duration with coronary heart disease in a large-scale rural population. *Sleep Medicine*.

[33] Sadabadi F, Darroudi S, Esmaily H, Asadi Z, Ferns GA, Mohammadpour AH (2023). The importance of sleep patterns in the incidence of coronary heart disease: a 6-year prospective study in Mashhad, Iran. *Scientific Reports*.

[34] Song C, Zhang R, Liao J, Fu R, Wang C, Liu Q (2020). Sleep quality and risk of coronary heart disease - a prospective cohort study from the English longitudinal study of ageing. *Aging*.

[35] Yang J, Wang K, Wang W, Niu J, Liu X, Shen H (2024). The Effect of Sleep Quality on Coronary Lesion Severity and Prognosis in the Young Acute Coronary Syndrome Population. *Journal of Cardiovascular Development and Disease*.

[36] Wang Z, Yang W, Li X, Qi X, Pan KY, Xu W (2022). Association of Sleep Duration, Napping, and Sleep Patterns With Risk of Cardiovascular Diseases: A Nationwide Twin Study. *Journal of the American Heart Association*.

[37] Bin YS (2016). Is Sleep Quality More Important Than Sleep Duration for Public Health?. *Sleep*.

[38] Yang TC, Park K (2015). To What Extent do Sleep Quality and Duration Mediate the Effect of Perceived Discrimination on Health? Evidence from Philadelphia. *Journal of Urban Health: Bulletin of the New York Academy of Medicine*.

[39] Buysse DJ, Reynolds CF, Monk TH, Berman SR, Kupfer DJ (1989). The Pittsburgh Sleep Quality Index: a new instrument for psychiatric practice and research. *Psychiatry Research*.

[40] Prati F, Romagnoli E, Gatto L, La Manna A, Burzotta F, Ozaki Y (2020). Relationship between coronary plaque morphology of the left anterior descending artery and 12 months clinical outcome: the CLIMA study. *European Heart Journal*.

[41] Hoevenaar-Blom MP, Spijkerman AMW, Kromhout D, van den Berg JF, Verschuren WMM (2011). Sleep duration and sleep quality in relation to 12-year cardiovascular disease incidence: the MORGEN study. *Sleep*.

[42] Ai S, Zhang J, Zhao G, Wang N, Li G, So HC (2021). Causal associations of short and long sleep durations with 12 cardiovascular diseases: linear and nonlinear Mendelian randomization analyses in UK Biobank. *European Heart Journal*.

[43] Irwin MR, Wang M, Campomayor CO, Collado-Hidalgo A, Cole S (2006). Sleep deprivation and activation of morning levels of cellular and genomic markers of inflammation. *Archives of Internal Medicine*.

[44] van Leeuwen WMA, Lehto M, Karisola P, Lindholm H, Luukkonen R, Sallinen M (2009). Sleep restriction increases the risk of developing cardiovascular diseases by augmenting proinflammatory responses through IL-17 and CRP. *PloS One*.

[45] Sang D, Lin K, Yang Y, Ran G, Li B, Chen C (2023). Prolonged sleep deprivation induces a cytokine-storm-like syndrome in mammals. *Cell*.

[46] Shah PK, Falk E, Badimon JJ, Fernandez-Ortiz A, Mailhac A, Villareal-Levy G (1995). Human monocyte-derived macrophages induce collagen breakdown in fibrous caps of atherosclerotic plaques. Potential role of matrix-degrading metalloproteinases and implications for plaque rupture. *Circulation*.

[47] Hansson GK, Libby P (2006). The immune response in atherosclerosis: a double-edged sword. *Nature Reviews. Immunology*.

[48] Atrooz F, Salim S (2020). Sleep deprivation, oxidative stress and inflammation. *Advances in Protein Chemistry and Structural Biology*.

[49] Vaccaro A, Kaplan Dor Y, Nambara K, Pollina EA, Lin C, Greenberg ME (2020). Sleep Loss Can Cause Death through Accumulation of Reactive Oxygen Species in the Gut. *Cell*.

[50] Shah R, Shah VK, Emin M, Gao S, Sampogna RV, Aggarwal B (2023). Mild sleep restriction increases endothelial oxidative stress in female persons. *Scientific Reports*.

[51] Boudjeltia KZ, Faraut B, Esposito MJ, Stenuit P, Dyzma M, Antwerpen PV (2011). Temporal dissociation between myeloperoxidase (MPO)-modified LDL and MPO elevations during chronic sleep restriction and recovery in healthy young men. *PloS One*.

[52] Fu X, Kassim SY, Parks WC, Heinecke JW (2001). Hypochlorous acid oxygenates the cysteine switch domain of pro-matrilysin (MMP-7). A mechanism for matrix metalloproteinase activation and atherosclerotic plaque rupture by myeloperoxidase. *The Journal of Biological Chemistry*.

[53] Kuo MY, Ou HC, Lee WJ, Kuo WW, Hwang LL, Song TY (2011). Ellagic acid inhibits oxidized low-density lipoprotein (OxLDL)-induced metalloproteinase (MMP) expression by modulating the protein kinase C-α/extracellular signal-regulated kinase/peroxisome proliferator-activated receptor γ/nuclear factor-κB (PKC-α/ERK/PPAR-γ/NF-κB) signaling pathway in endothelial cells. *Journal of Agricultural and Food Chemistry*.

[54] Meng L, Zheng Y, Hui R (2013). The relationship of sleep duration and insomnia to risk of hypertension incidence: a meta-analysis of prospective cohort studies. *Hypertension Research: Official Journal of the Japanese Society of Hypertension*.

[55] Garg H (2018). Role of optimum diagnosis and treatment of insomnia in patients with hypertension and diabetes: A review. *Journal of Family Medicine and Primary Care*.

[56] Cappuccio FP, D’Elia L, Strazzullo P, Miller MA (2010). Quantity and quality of sleep and incidence of type 2 diabetes: a systematic review and meta-analysis. *Diabetes Care*.

[57] Frøjd LA, Munkhaugen J, Moum T, Sverre E, Nordhus IH, Papageorgiou C (2021). Insomnia in patients with coronary heart disease: prevalence and correlates. *Journal of Clinical Sleep Medicine: JCSM: Official Publication of the American Academy of Sleep Medicine*.

[58] Matsuda R, Kohno T, Kohsaka S, Fukuoka R, Maekawa Y, Sano M (2017). The prevalence of poor sleep quality and its association with depression and anxiety scores in patients admitted for cardiovascular disease: A cross-sectional designed study. *International Journal of Cardiology*.

[59] Liu T, Ji H, Jian X, Wang W, Fan Z (2022). Novel nomogram for predicting coronary vulnerable plaque risk in patients with coronary artery disease. *Biomarkers in Medicine*.

[60] Abtahian F, Yonetsu T, Kato K, Jia H, Vergallo R, Tian J (2014). Comparison by optical coherence tomography of the frequency of lipid coronary plaques in current smokers, former smokers, and nonsmokers. *The American Journal of Cardiology*.

[61] Wang Y, Zhao Z, Gao X, Li L, Liu G, Chen W (2016). Type D Personality and Coronary Plaque Vulnerability in Patients With Coronary Artery Disease: An Optical Coherence Tomography Study. *Psychosomatic Medicine*.

